# Local Environmental Conditions Promote High Turnover Diversity of Benthic Deep-Sea Fungi in the Ross Sea (Antarctica)

**DOI:** 10.3390/jof8010065

**Published:** 2022-01-08

**Authors:** Giulio Barone, Cinzia Corinaldesi, Eugenio Rastelli, Michael Tangherlini, Stefano Varrella, Roberto Danovaro, Antonio Dell’Anno

**Affiliations:** 1 Institute for Marine Biological Resources and Biotechnology, National Research Council, Largo Fiera della Pesca 2, 60125 Ancona, Italy; giulio.barone@irbim.cnr.it; 2 Department of Life and Environmental Sciences, Polytechnic University of Marche, Via Brecce Bianche, 60131 Ancona, Italy; r.danovaro@univpm.it; 3 Department of Materials, Environmental Sciences and Urban Planning, Polytechnic University of Marche, Via Brecce Bianche, 60131 Ancona, Italy; c.corinaldesi@staff.univpm.it (C.C.); s.varrella@univpm.it (S.V.); 4 Department of Marine Biotechnology, Stazione Zoologica Anton Dohrn, Fano Marine Centre, Viale Adriatico 1-N, 61032 Fano, Italy; eugenio.rastelli@szn.it; 5 Department of Research Infrastructures for Marine Biological Resources, Stazione Zoologica Anton Dohrn, Fano Marine Centre, Viale Adriatico 1-N, 61032 Fano, Italy; michael.tangherlini@szn.it; 6 Stazione Zoologica Anton Dohrn, Villa Comunale, 80121 Naples, Italy

**Keywords:** deep-sea sediments, fungal diversity, trophic conditions, Antarctica, Ross Sea

## Abstract

Fungi are a ubiquitous component of marine systems, but their quantitative relevance, biodiversity and ecological role in benthic deep-sea ecosystems remain largely unexplored. In this study, we investigated fungal abundance, diversity and assemblage composition in two benthic deep-sea sites of the Ross Sea (Southern Ocean, Antarctica), characterized by different environmental conditions (i.e., temperature, salinity, trophic availability). Our results indicate that fungal abundance (estimated as the number of 18S rDNA copies g^−1^) varied by almost one order of magnitude between the two benthic sites, consistently with changes in sediment characteristics and trophic availability. The highest fungal richness (in terms of Amplicon Sequence Variants−ASVs) was encountered in the sediments characterized by the highest organic matter content, indicating potential control of trophic availability on fungal diversity. The composition of fungal assemblages was highly diverse between sites and within each site (similarity less than 10%), suggesting that differences in environmental and ecological characteristics occurring even at a small spatial scale can promote high turnover diversity. Overall, this study provides new insights on the factors influencing the abundance and diversity of benthic deep-sea fungi inhabiting the Ross Sea, and also paves the way for a better understanding of the potential responses of benthic deep-sea fungi inhabiting Antarctic ecosystems in light of current and future climate changes.

## 1. Introduction

The Southern Ocean, surrounding Antarctica, plays a key role in global ocean circulation and biogeochemical cycles [1,2]. Here, primary productivity and carbon export to the seafloor are highly variable in space and time, with the highest rates of primary production occurring during the austral summer in the coastal polynyas (regions of open water surrounded by sea ice; [3,4]), marginal ice zone [5] and continental shelf [6,7,8]. Despite the extreme environmental conditions (e.g., low temperature, highly variable nutrient availability), the Southern Ocean hosts rich and diverse benthic deep-sea assemblages, including several endemic species [9,10].

Microbial assemblages in benthic deep-sea ecosystems play an important role in C and nutrient cycling and transfer of energy and material to the higher trophic levels [11]. Besides prokaryotes, fungi are widespread in deep-sea environments spanning from hypersaline anoxic basins [12,13,14] to cold seeps [15,16], from hydrothermal vents [17,18,19] to surface and subsurface sediments [13,20,21,22,23,24], including benthic Antarctic systems [25,26,27,28] and references therein). Theoretical estimates suggest that fungi can be the most diversified component of eukaryotes on Earth, with more than 5 million species of which only 5% have been described [29,30]. This gap applies in particular to deep-sea ecosystems, where a significant fraction of fungal diversity is still unknown [24,31,32]. Recent studies suggest that a variety of environmental factors (e.g., temperature, salinity, nutrient availability) can influence the diversity and assemblage composition of fungi in marine ecosystems [33,34]. However, factors controlling the distribution and diversity of fungi in benthic deep-sea ecosystems remain largely unexplored to date [24] and even less is known of fungi inhabiting the Southern Ocean. In deep-sea ecosystems, fungi are not only highly diversified, but they are likely involved in the degradation and cycling of organic matter [13,18,35,36,37,38]. In benthic deep-sea ecosystems, organic matter mainly consists of refractory organic compounds [39,40], and fungi are known to be efficient degraders of complex organic molecules not suitable for other heterotrophic microbes [41,42]. However, their role in C and nutrient cycling in benthic deep-sea ecosystems remains poorly understood [24]. 

Global climate change is altering marine biodiversity and food web dynamics, and such effects are particularly pronounced at high latitudes [43,44,45]. Changes in environmental conditions (e.g., temperature, salinity, nutrient availability) due to climate changes in polar regions can induce a domino effect that could impact biodiversity and ecosystem functioning from the continental shelf down to the deep seafloor [46,47]. Nevertheless, information on benthic deep-sea fungal assemblages of Antarctic ecosystems is scant and insufficient to understand and predict how these components will respond to the expected changes in environmental conditions.

In this study, we investigated the abundance, diversity and assemblage composition of fungi in two benthic deep-sea sites of the Ross Sea (one of the most productive sectors of the whole Southern Ocean; [48]), characterized by different environmental conditions in terms of trophic availability and thermohaline regime. This work aims at shedding light on the ecology of benthic deep-sea fungi and factors shaping their distribution at different spatial scales (i.e., between stations of the same site and between sites). This information is crucial for better comprehension of the responses of benthic fungal assemblages inhabiting Antarctic ecosystems, and also in light of climate change scenarios. 

## 2. Materials and Methods

### 2.1. Study Area and Sampling Strategy

**Figure 1:**
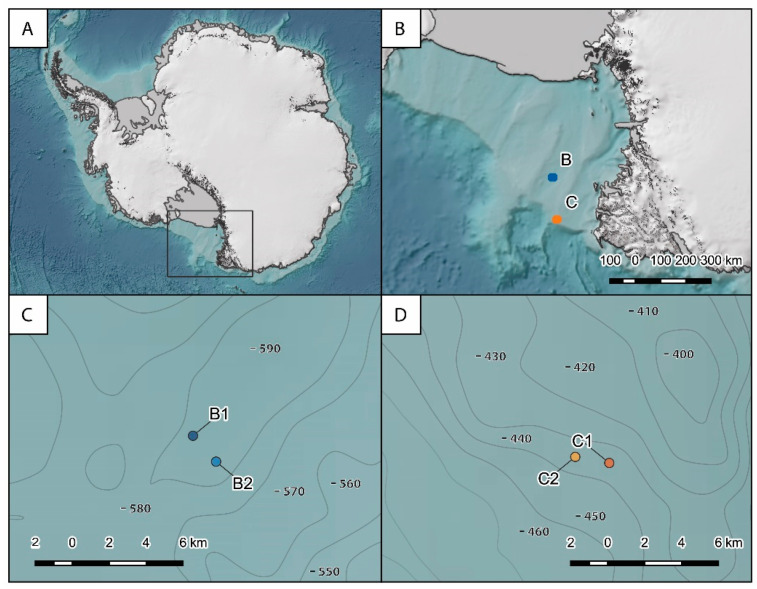
Location of the study area (**A**) and sampling locations of the two benthic deep-sea sites investigated (**B**) and of the stations within sites B (**C**) and C (**D**). The map was generated upon freely available layers within QGIS 3.22 environment (http://www.qgis.org; accessed on 7 January 2022).

Sediment samples were collected in the Ross Sea, Southern Ocean (Figure 1), during the austral summer 2017 onboard the research vessel *M/N Italica* in the framework of the XXXII Italian Antarctic Expedition. Samples were collected at two different sites, named B and C, located about 170 km from each other. Site B (average depth of ca. 580 m) is located in the cross-shelf valley in the northern part of the Joides basin, and it is characterized by bio-siliceous olive-gray mud sediments. Site C is located at ca. 430 m depth close to the shelf break on the northern flank of the Mawson Bank, and it is characterized by sand, gravel, pebbles and coarse biogenic carbonate debris and high near-bottom current velocities (up to 20 cm s^−1^; [49]). At both sites, two stations located at ca. 2 km from each other were selected to investigate spatial variability within the same site (hereafter defined B1 and B2 and C1 and C2). At each station, the main physical–chemical characteristics of the bottom waters were acquired by CTD casts along with the collection of undisturbed sediment samples by three independent box corer deployments. Once on board, sediment subsamples of the top 1 cm were collected and stored at −20 °C until laboratory analyses for the determination of organic matter quantity and quality (used as a proxy of trophic conditions [50,51]), fungal abundance, diversity and assemblage composition. All samples were processed within six months of their collection.

### 2.2. Trophic Conditions

Trophic conditions of benthic systems were assessed on the basis of the quantity and biochemical composition of organic matter [50,51]. Chloroplastic pigments (chlorophyll-a and phaeopigments) were analyzed fluorometrically [52]. Pigments were extracted with 90% acetone (12 h in the dark at 4 °C). After centrifugation, the supernatant was used to determine the functional chlorophyll-a and then acidified with 0.1N HCl to estimate phaeopigments. Total phytopigment concentration (CPE) was defined as the sum of chlorophyll-a and phaeopigment concentrations.

Protein, carbohydrate and lipid concentrations in the sediment were determined according to previously described protocols [52]. Briefly, protein concentration was assessed by a colorimetric method, based on the reaction of proteins with copper tartrate and the Folin–Ciocalteau in a basic environment (pH 10), which provides a stable blue coloration with an intensity proportional to protein concentration. Carbohydrate concentration was determined spectrophotometrically based on the reaction between carbohydrates and phenol in the presence of sulfuric acid, which provides a coloration whose intensity is proportional to carbohydrate concentration. Lipids were extracted by direct elution with chloroform and methanol followed by reaction with sulfuric acid and determination by a colorimetric method. Protein, carbohydrate and lipid concentrations were expressed as albumin, glucose and tripalmitin equivalents, respectively. All analyses were carried out in three replicates. Protein, carbohydrate and lipid concentrations were converted to carbon equivalents (conversion factors: 0.49, 0.40 and 0.75 gC g^−1^, respectively) to determine biopolymeric C content (BPC) in the sediments [50]. The protein to carbohydrate ratio (P:C) was used as a proxy of organic matter quality [51].

### 2.3. DNA Extraction and Purification for Molecular Analysis

DNA was extracted and purified from sediment samples using PowerSoil DNA isolation kit (QIAGEN), following the manufacturer’s instruction with slight modifications to remove extracellular DNA (based on three subsequent washing steps), before DNA extraction [52].

### 2.4. Fungal Abundance Estimated by Quantitative Real-Time PCR (qPCR)

To estimate fungal abundance, DNA aliquots were used for quantitative real-time PCR (qPCR) analysis of the fungal-specific 18S rRNA gene [53]. Briefly, fungi-specific primers (FR1 5′-AIC CAT TCA ATC GGT AIT-3′) and FF390 (5′-CGA TAA CGA ACG AGA CCT-3′), which amplify a 18S rRNA gene fragment of about 350 bp [54], were used with the Sensi-FAST SYBR Q-PCR kit (Bioline, London, UK). The 15 μL reactions contained 8 µl Sensi-FAST master mix, 1 μL of each primer (final concentration 1 μM), 1μL of DNA template and 5 μL nuclease-free molecular-grade water [53]. A Bio-Rad iQ5 instrument was used to perform qPCR analyses using the following thermal protocol: 94 °C for 3 min., then 40 cycles of 94 °C for 10 s, annealing at 50 °C for 15 s, elongation at 72 °C for 20 s and acquisition of fluorescence data at 82 °C. The CFX Manager™ (v3.1) software was used to calculate Cq, efficiency (E) and R^2^ values of standard curves for each plate and to quantify 18S rDNA copy numbers present in the samples analyzed. Standard curves were generated using known concentrations of *Aspergillus niger* 18S rDNA. The number of fungal 18S rDNA copies was standardized per gram of dry sediment.

### 2.5. Fungal Diversity and Assemblage Composition

DNA extracted from two sediment samples collected at each station by independent box corer deployments was amplified using the primer set ITS1F (5′-GGAAGTAAAAGTCGTAACAAGG-3′) and ITS2 (5′- GCTGCGTTCTTCATCGATGC-3′), which amplify the internal transcribed spacer-1 (ITS1) region of the fungal rRNA gene [55,56]. Amplicons were sequenced on an Illumina MiSeq platform by the LGC group (Berlin, Germany), following the Earth Microbiome Project protocols (http://www.earthmicrobiome.org/emp-standard-protocols/; accessed on 15 September 2018). Paired-end sequences were analyzed within the QIIME2 environment [57]. First, the ITSxpress plugin was used to trim sequences targeting the ITS1 region [58]; then, trimmed paired-end sequences were analyzed through the DADA2 procedure [59], and the resulting biologically significant Amplicon Sequence Variants (ASVs) were compared against the UNITE database (Version: 8.3; Last updated: 11 December 2020) for taxonomic affiliation [58]. Taxonomic affiliation was performed through the USEARCH SINTAX procedure [60] using three different thresholds: 0.8 (default), 0.6 and 0.5 to evaluate potential distant affiliations. To allow for a proper comparison among samples, the ASV table was then rarefied to 900 randomly selected sequences, corresponding to the lowest read count obtained in our samples [61,62].

### 2.6. Data Analyses

Differences in environmental and trophic variables, fungal abundance (as 18S rDNA copy number) and ASV richness between and within sites were tested by permutational two-way nested analysis of variance (2-way nested PERMANOVA; [63,64], considering the two factors Site (2 levels: B and C) and Station (nested in Site, 2 levels: 1 and 2). P-values were calculated with unrestricted permutation of raw data (perm.: 9999) with adonis function in *vegan* package. To investigate the relationships between fungal abundance and ASV richness and environmental and trophic variables, Spearman Rank correlation analyses were carried out.

The rarefied ASV table was used to assess the number of either “core” ASVs (i.e., at least one ASV present in all samples) and “exclusive” ASVs (i.e., ASVs found only in a single sample) and the output was visualized by network analysis through the Gephi package [65]. To determine similarities of the fungal assemblage composition between stations and sites, a similarity percentage analysis (SIMPER; [66]) was carried out. To identify potential factors influencing fungal assemblage composition, DistLM routine and distance-based redundancy analyses (dbRDA) were carried out with Primer+PERMANOVA (v7) [67]. In particular, temperature, salinity and dissolved oxygen concentrations of the bottom waters and quantity (i.e., biopolymeric C concentrations and total phytopigment content) and quality (protein to carbohydrate ratio) of organic matter in the sediments were used as predictor variables.

## 3. Results and Discussion

The thermohaline conditions of bottom waters of the benthic systems investigated in the present study changed widely, with temperature values ranging from −1.880 °C to −0.046 °C and salinity values ranging from 34.650 to 34.756 (Table 1). Stations at Site B were characterized by colder, saltier and more oxygenated waters than stations at Site C, reflecting differences in water mass characteristics. In particular, cold waters with temperatures below the surface freezing point observed at stations of Site B were associated with the Ice Shelf Water (ISW) overflowing on the continental slope of the Ross Sea [68]. The two benthic sites were also characterized by differences in terms of sediment grain size, which was mainly represented by silt–clay particles at stations of Site B and by coarse particles, including carbonate debris, at stations of Site C.

There is evidence that the distribution and accumulation of organic matter in surface sediments of deep-sea systems of the Ross Sea are influenced not only by vertical inputs from the upper water column, but also by lateral advection processes [69,70]. The formation and cascading of the Ice Shelf Water can represent an important process of C supply to the sea bottom, which may be responsible for the high organic matter content observed at the benthic deep-sea stations of Site B (Table 1). Values of total phytopigment concentrations, a proxy of the organic material produced by photosynthesis in surface waters and settling on the seafloor, and biopolymeric C concentrations in surface sediments of stations of Site B were, indeed, 10 times higher than those of Site C stations (Table 1).

**Figure 2:**
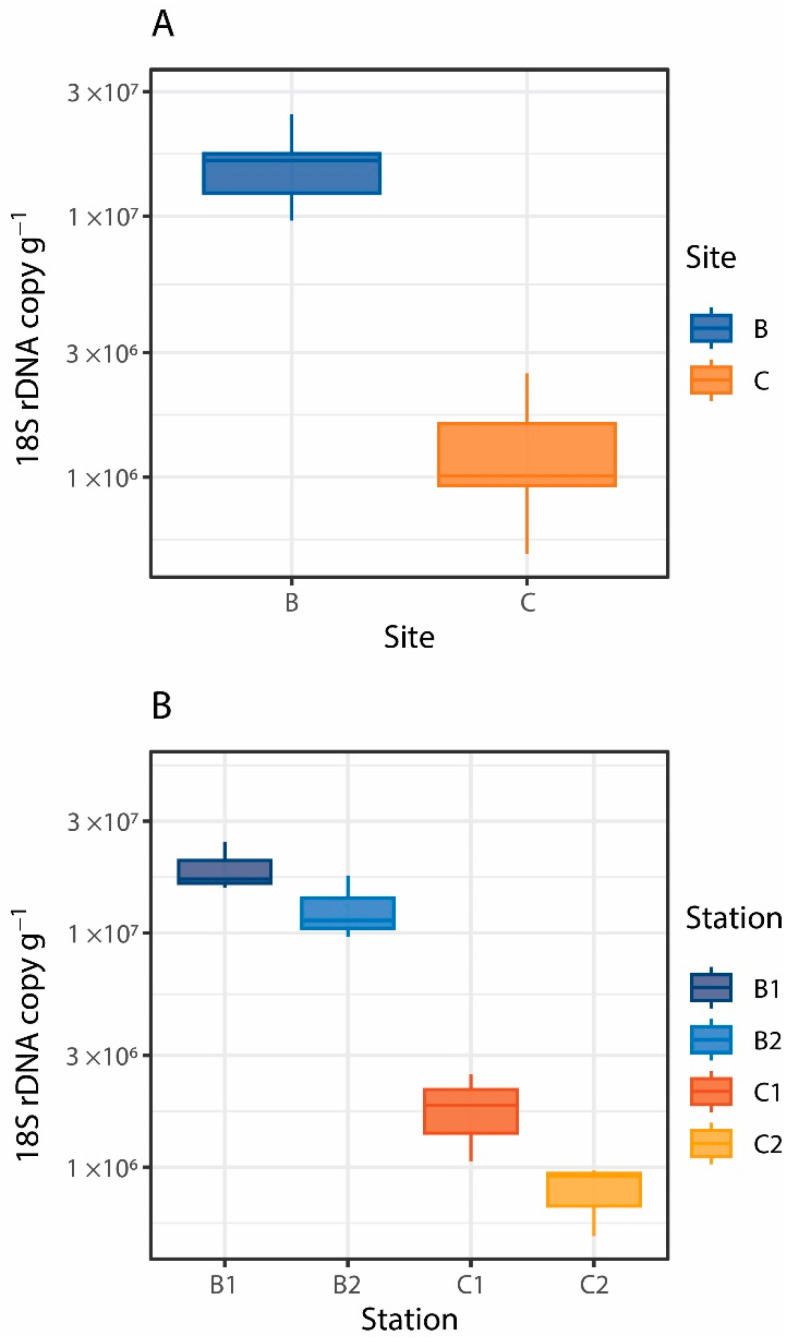
Boxplots showing fungal abundance, expressed as the number of 18S rDNA copies per gram of dry sediment, of the two benthic deep-sea sites investigated (**A**) and of the stations within sites B and C (**B**).

**Figure 3:**
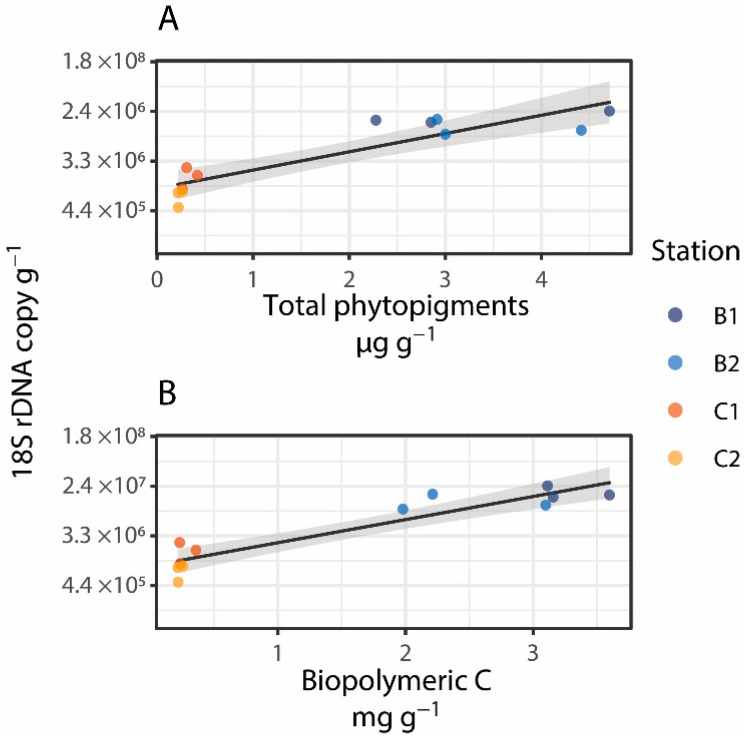
Relationships between fungal abundance (expressed as the number of 18S rDNA copies per gram of dry sediment) and total phytopigment (**A**) and biopolymeric C (**B**) concentrations in the study area. Gray shade represents the 95% confidence interval of the linear model interpolating the observations.

There is a general consensus that trophic availability controls the abundance and distribution of benthic deep-sea standing stocks from prokaryotes to meio- and macrofauna [71,72], but information on its relevance in influencing the distribution of deep-sea fungi is still largely lacking [24]. In this study, fungal abundance, expressed as fungal 18S rDNA copies per gram of dry sediment, ranged from 1.3 ± 0.7 × 10^6^ copies g^−1^ to 1.6 ± 0.5 × 10^7^ copies g^−1^ (Figure 2A,B). Our results fall within previously reported ranges for deep-sea sediments of the Pacific Ocean (from 3.5 × 10^6^ to 5.2 × 10^7^ 28S rDNA copies g^−1^ [73]) and the Mediterranean Sea (from 1.4 × 10^6^ to 5.1 × 10^7^ 18S rDNA copies g^−1^ [24]) and provide the first evidence of the quantitative importance of fungi in benthic deep-sea ecosystems of the Southern Ocean. Fungal abundance changed between and within sites (Site: Pseudo-F_2,8_ = 5.27, *p* < 0.05; Station (Site): F_1,8_ = 15.86, *p* < 0.05; Figure 2A,B), with values up to 1 order of magnitude higher at stations of Site B than at stations of Site C. Significant positive relationships were found between fungal abundance and total phytopigment and biopolymeric C concentrations in the sediment (Figure 3A,B). In particular, biopolymeric C concentrations alone explained 87% of the total variation in fungal abundance (t = 8.1, *p* < 0.001, Spearman’s ρ = 0.87). Overall, these findings suggest that benthic deep-sea fungi, besides prokaryotes, can be actively involved in the decomposition and utilization of organic matter settling on the seafloor, thus contributing to its cycling.

**Figure 4:**
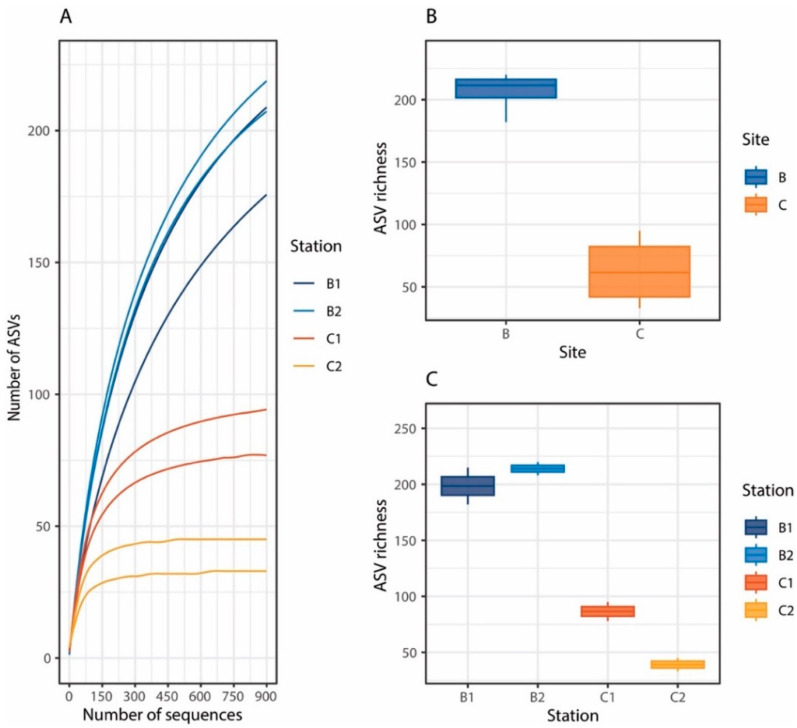
(**A**) Rarefaction curves from random subsampling (100 iterations) of 900 reads corresponding to the lowest read number obtained from the different sediment samples analyzed; (**B**) boxplot showing the fungal ASV richness in the two benthic deep-sea sites investigated, and (**C**) boxplot showing the fungal ASV richness in the four benthic deep-sea stations investigated.

The clustering of the 113,635 fungal ITS sequences obtained in the present study after trimming allowed us to identify a total of 1251 fungal ASVs. Rarefaction curves indicated that the sequencing effort was sufficient to describe the fungal diversity present in the benthic systems investigated, even after rarefaction to the lowest sequencing depth (Figure 4A). We found a high variability of fungal ASV richness between the two sites (Figure 4B), and also between the stations, in particular of Site C (Figure 4C). Fungal ASV richness was higher at Site B, where a higher trophic availability was also found, compared to Site C (F_1,4_ = 108.2; *p* < 0.01). However, the values of fungal richness reported in the present study fell within the range previously reported for other benthic deep-sea ecosystems [22,24], including Southern Ocean sediments [26].

**Figure 5:**
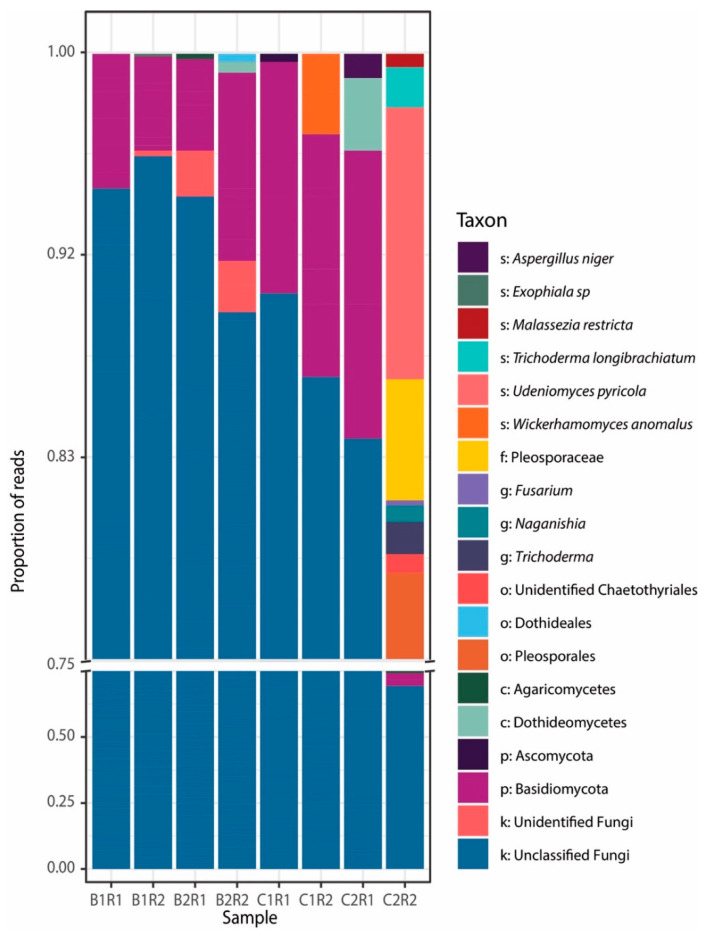
Stacked bar plot showing the relative abundance of fungal taxa at the lowest taxonomic levels identified in each deep-sea sediment sample collected in the Ross Sea. Taxonomic affiliation was assigned using a confidence threshold cutoff of 0.8.

Taxonomic analysis showed that using a default confidence threshold (cutoff of 0.8), most of the fungal ASVs could not be assigned to known fungal taxa (on average ca. 90%, Figure 5 and Figure S1). Relaxing the confidence thresholds, the number of unknown fungal ASVs decreased (68% with a 0.6 cutoff and 58% with a 0.5 cutoff), but with a less-reliable classification (Figure S1). This result indicates that benthic deep-sea Antarctic sediments can harbor a large number of novel fungal lineages, while fungal ASVs affiliating to known fungal taxa included members affiliated to Ascomycota and Basidiomycota, which typically represent the main phyla reported in different benthic deep-sea ecosystems worldwide [24,26,27,32].

Only a few ASVs could be affiliated to known fungal genera. In particular, we found 12 ASVs affiliating to nine genera, including genera commonly encountered in a variety of benthic deep-sea ecosystems (e.g., *Aspergillus*; [24,32]) and polar systems (e.g., *Naganishia*, *Dothideomycetes* and *Agaricomycetes*; [27,74,75]). Fungi belonging to the genera *Trichoderma* found in this study were already reported and isolated from lake and sediments of the Penguin Island in Antarctica [76,77], while other genera commonly found in Antarctic sediments, such as *Metschnikowia*, *Galciozyma* and *Psedogymnoascus*, were not encountered (for a more detailed list see [28]). Furthermore, other fungal taxa, including members belonging to *Fusarium* and *Wickerhamomyces*, have been reported to be associated with Antarctic sponges and macroalgae [78,79,80], while taxa affiliated with *Exophiala* and *Aspergillus* have been previously isolated from different Antarctic marine samples [28,81]. Such a comparison suggests that Antarctic deep-sea sediments can host profoundly different fungal assemblages depending on specific environmental and ecological settings.

**Figure 6:**
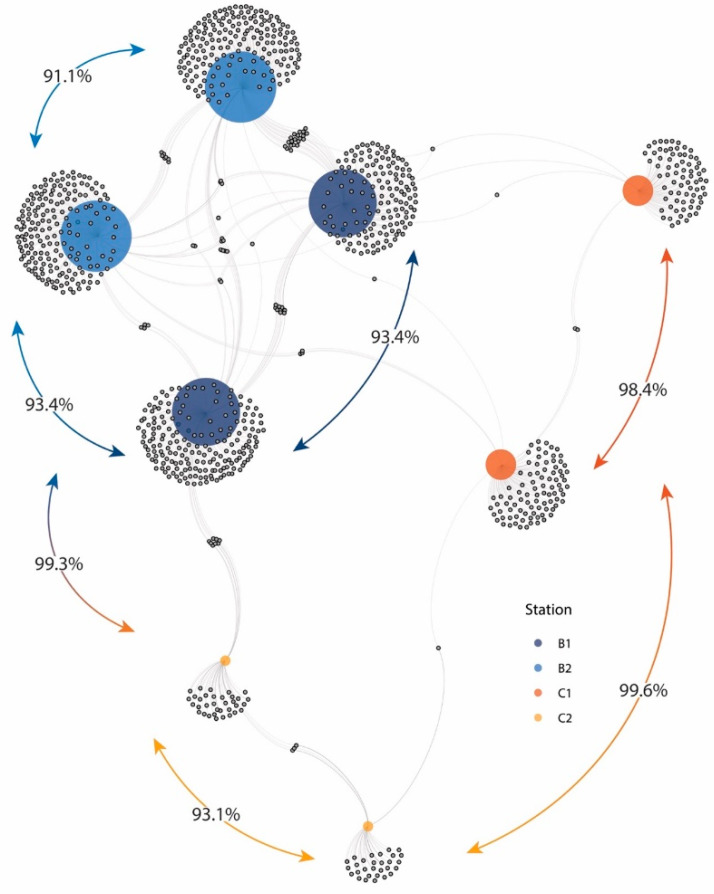
Network plot based on fungal assemblage composition found in the different sediment samples analyzed. Colored nodes represent sampling stations, while gray nodes represent the ASVs belonging to one or more samples to which are connected by gray edges. Arrows connecting samples indicate the Bray–Curtis dissimilarity calculated upon the log-transformed rarefied ASV table.

SIMPER analysis revealed a very low similarity between the fungal assemblage compositions of the two sites and within them, as highlighted by the network plot (Figure 6). In particular, the average similarity between stations of Site B were higher than those between stations of Site C (7.7 vs. 1.6%), while the similarity between Site B and C was on average < 1%. Such very low similarity values were due to the presence of a large fraction of exclusive ASVs of each sample (accounting for 76–94% of the total ASVs; Figure 6). Overall, these findings suggest that differences in ecological and environmental conditions occurring even at spatial scales of a few meters (i.e., between replicates) can have a major role in shaping fungal assemblage composition, thus contributing to increase fungal turnover diversity.

**Figure 7:**
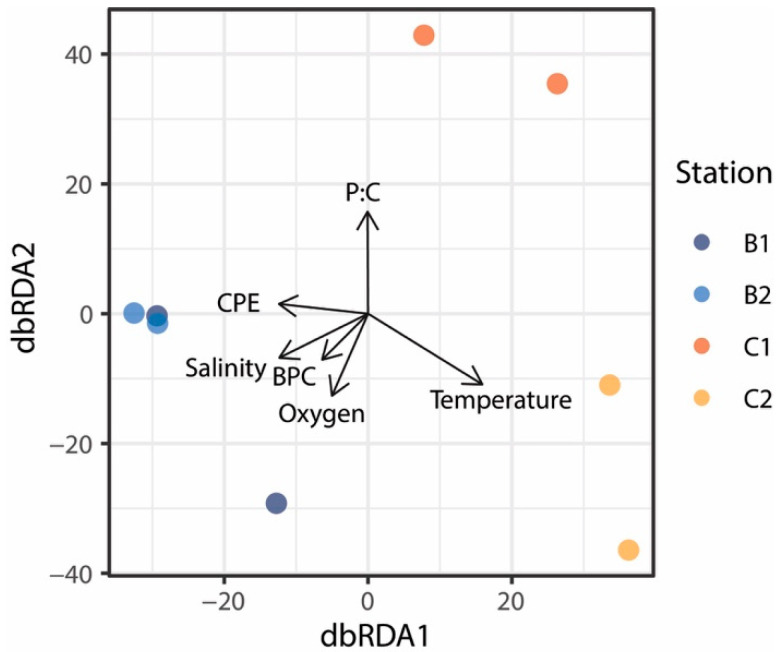
Output of the distance-based redundancy analysis (dbRDA) showing the relationships between the fungal assemblage composition and environmental and trophic variables analyzed in this study.

Previous studies suggested that environmental factors and trophic availability can influence fungal assemblage composition [24,82,83,84]. The distance-based redundancy analysis (dbRDA) allowed us to identify significant relationships between fungal assemblage composition and trophic (total phytopigment and biopolymeric C concentrations, protein to carbohydrate ratio) and environmental variables (temperature, salinity and oxygen concentrations). Altogether, the environmental and trophic variables explained 87% of the observed variation in fungal assemblage composition, but only temperature significantly explained 18% of the total variance (Figure 7). Thus, other factors acting at the local scale, such as habitat heterogeneity, competition and predation processes [85], may have an additional role in promoting a high diversification of benthic deep-sea fungi. Overall, results of the present study indicate that Antarctic deep-sea sediments host abundant and highly diversified fungal assemblages most of which still unidentified and suggest that fungi inhabiting Antarctic benthic deep-sea ecosystems can be sensitive to an interplay of environmental and ecological factors, whose variations, potentially induced also by climate changes, can profoundly influence their assemblage composition.

## 4. Conclusions

This study provides new insights into the quantitative relevance and diversity of benthic deep-sea fungi in the Ross Sea. Our findings reveal that the distribution of fungal abundance and richness is primarily driven by trophic availability, whereas an interplay of factors shapes fungal assemblage composition. Our findings also suggest that the spatial variability even at a small scale can promote important differences in deep-sea fungal assemblages, thus allowing for the maintenance of overall high fungal diversity. Results reported in this study could be relevant for a better understanding of the potential impact of thermohaline and trophic modifications due to climate changes on Antarctic deep-sea ecosystems. Modifications of ice coverage and thermohaline conditions affecting the planktonic food web structure could, indeed, profoundly influence organic carbon export to the seafloor, with cascading effects on benthic deep-sea biodiversity and ecosystem functioning. Although altered freezing and melting cycles of Antarctic pack ice are expected to drastically change ecosystem functioning, we still have a limited knowledge of biogeochemical cycles and ecological processes in which fungi are involved. Therefore, our results highlight the need to improve our understanding of the ecological role of benthic deep-sea fungi for better comprehension and prediction of the potential effects of climate changes on Antarctic deep-sea ecosystem functioning.

## Figures and Tables

**Table 1:** Values of temperature, salinity and dissolved oxygen concentrations of the bottom waters and biopolymeric carbon and total phytopigment concentrations and protein to carbohydrate ratios (P:C) in the surface sediments of the different stations investigated. Mean values and standard deviations (±) are reported.

Site	Station	Depth	Latitude	Longitude	Temperature	Salinity	Dissolved Oxygen
		(m)	EPSG: 4326	EPSG: 4326	(°C)		(mg L^−1^)
B	B1	585	−74.03951	175.07945	−1.88 ± 0.0002	34.752 ± 0.0002	10.96 ± 0.05
B	B2	587	−74.01603	175.04279	−1.878 ± 0.0002	34.756 ± 0.0001	11 ± 0.05
C	C1	433	−72.49527	174.94336	−0.5 ± 0.001	34.65 ± 0.0002	6.25 ± 0.003
C	C2	434	−72.49967	174.99696	−0.046 ± 0.0024	34.667 ± 0.0001	9.92 ± 0.005
		**Biopolymeric Carbon**		**Total phytopigments**		**P:C**	
		**(mg g** **^−1^)**		**(µg g** **^−1^)**			
B	B1	3.23 ± 0.23		3.28 ± 1.27		0.39 ± 0.1	
B	B2	2.32 ± 0.61		3.44 ± 0.84		0.19 ± 0.1	
C	C1	0.28 ± 0.08		0.33 ± 0.08		0.7 ± 0.19	
C	C2	0.23 ± 0.08		0.24 ± 0.11		0.46 ± 0.06	

## Data Availability

Raw sequencing reads have been deposited in the NCBI Sequence Read Archive (SRA) under the accession numbers PRJNA791191.

## Supplementary Materials

The following supporting information can be downloaded at: https://www.mdpi.com/article/10.3390/jof8010065/s1: Figure S1. Taxonomic analysis of fungal ASVs obtained using 3 different confidence thresholds through the USEARCH SINTAX command.
