# Supplementary material for: Local Environmental Conditions Promote High Turnover Diversity of Benthic Deep-Sea Fungi in the Ross Sea (Antarctica)
Source: J Fungi (Basel). 2022 Jan 8;8(1):65. doi: 10.3390/jof8010065 (PMC8781733; doi:10.3390/jof8010065)
Supplement: Supplementary file 1 [file jof-08-00065-s001.zip › jof-1541419-supplementary.pdf]

Supplementary figure

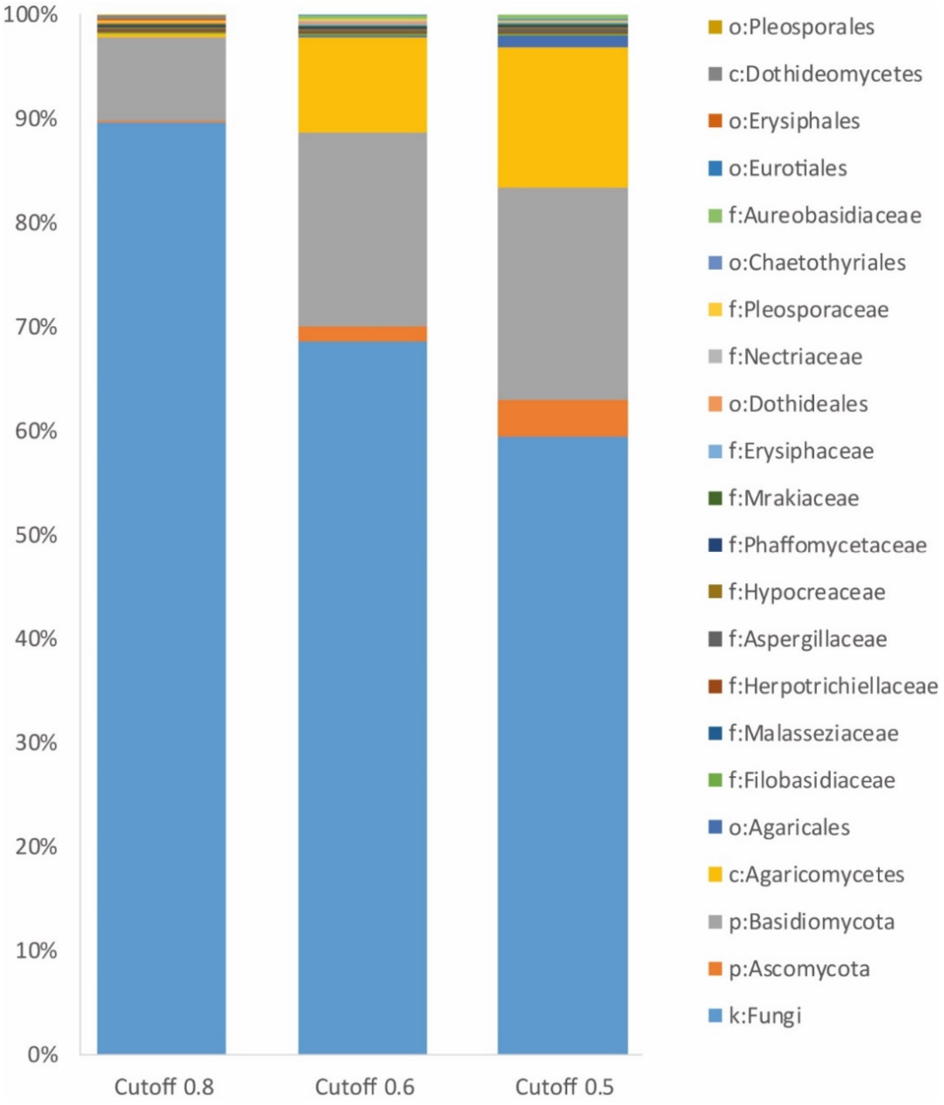

Figure S1. Taxonomic analysis of fungal ASVs obtained using 3 different confidence thresholds through the USEARCH SINTAX command.
